# A Bioinspired Cellulosic Elastomer with Ultrahigh Robustness Enabled by Structure-Induced Reconstruction for Impact Protection

**DOI:** 10.34133/research.1460

**Published:** 2026-10-08

**Authors:** Geyuan Jiang, Minxin Wang, Guangwen Xu, Dawei Zhao, Haipeng Yu

**Affiliations:** ^1^ School of Materials Science and Engineering, Shenyang University of Technology, Shenyang, Liaoning, China.; ^2^ Key Laboratory on Resources Chemicals and Materials of Ministry of Education, Shenyang University of Chemical Technology, Shenyang, Liaoning, China.; ^3^ Key Laboratory of Bio-based Material Science and Technology of Ministry of Education, Northeast Forestry University, Harbin, Heilongjiang, China.

## Abstract

Elastomers that combine high strength, exceptional toughness, self-healing capability, and scalable processability remain difficult to achieve. Here, we report a leech-inspired cellulosic elastomer constructed from cellulose, diatomite, and polyacrylamide through ethanol-induced structural reconstruction. The resulting bioinspired elastomer exhibits enhanced mechanical performance and efficient energy dissipation, with nearly complete compressive recoverability, a compressive strength above 90 MPa, and a load-bearing capacity exceeding 160,000 times its own weight. The elastomer further demonstrates high resistance to static puncture and dynamic impact, with a puncture resistance energy of 304.22 mJ and an impact toughness of 28.62 MJ m^−3^, while maintaining self-healing capability without thermal treatment and tolerance to ultralow temperatures down to −196 °C. In addition, the elastomer is compatible with roll-to-roll manufacturing and can be produced at the meter scale at a low cost. This work offers a scalable strategy for fabricating robust elastomers with potential applications in advanced impact protection and emerging soft technologies.

## Introduction

Nature’s biological systems, with their highly optimized and sophisticated structural organizations, offer valuable inspiration for the design of functional materials [1–3]. In particular, biological tissues often achieve a balance between strength, toughness, and energy dissipation through the synergistic integration of rigid and flexible components across multiple scales [4–6]. For example, marine shells, leech tissues, and the exoskeletons of ironclad beetles achieve high mechanical robustness through such integrated designs, offering valuable strategies for developing high-performance synthetic materials [7–12].

Elastomers, as soft synthetic materials, have been widely applied in protective equipment, intelligent robotics, and wearable electronics [13–17]. However, their production typically requires high-temperature and high-pressure conditions as well as blending-vulcanization procedures, which are energy intensive and operationally complex [18–21]. Moreover, the lack of effective energy-dissipation mechanisms often limits their mechanical performance [22–24]. A central challenge in the design of robust elastomers is overcoming the trade-off between strength and toughness. Increasing strength often leads to brittleness, whereas introducing soft dissipative components to improve toughness can compromise structural integrity and mechanical strength [25,26]. These challenges also hinder the development of high-performance elastomers using biomass feedstocks.

Among various biological tissues, leeches provide an attractive model for the design of robust elastomers because their soft bodies exhibit extraordinary tissue strength and structural toughness, sustaining loads exceeding 10,000 times their body weight [27,28]. This mechanical resilience arises from the synergistic architecture of muscle fibers, muscle proteins, and muscular tissues. Under external loading, longitudinal muscle fibers deform to efficiently transmit stress and enhance load-bearing capacity (Fig. 1A). Muscle proteins further accommodate microscale deformation and disperse local stress, thereby preserving structural integrity. In addition, the interconnected muscular tissues absorb and store impact energy through elastic deformation, enabling rapid recovery after unloading. The synergistic function of these components enables efficient stress transfer, deformation accommodation, and energy dissipation, allowing leeches to maintain structural integrity under extreme external loading. These mechanical characteristics provide an appealing biological model for the design of robust elastomers.

**Fig. 1.**
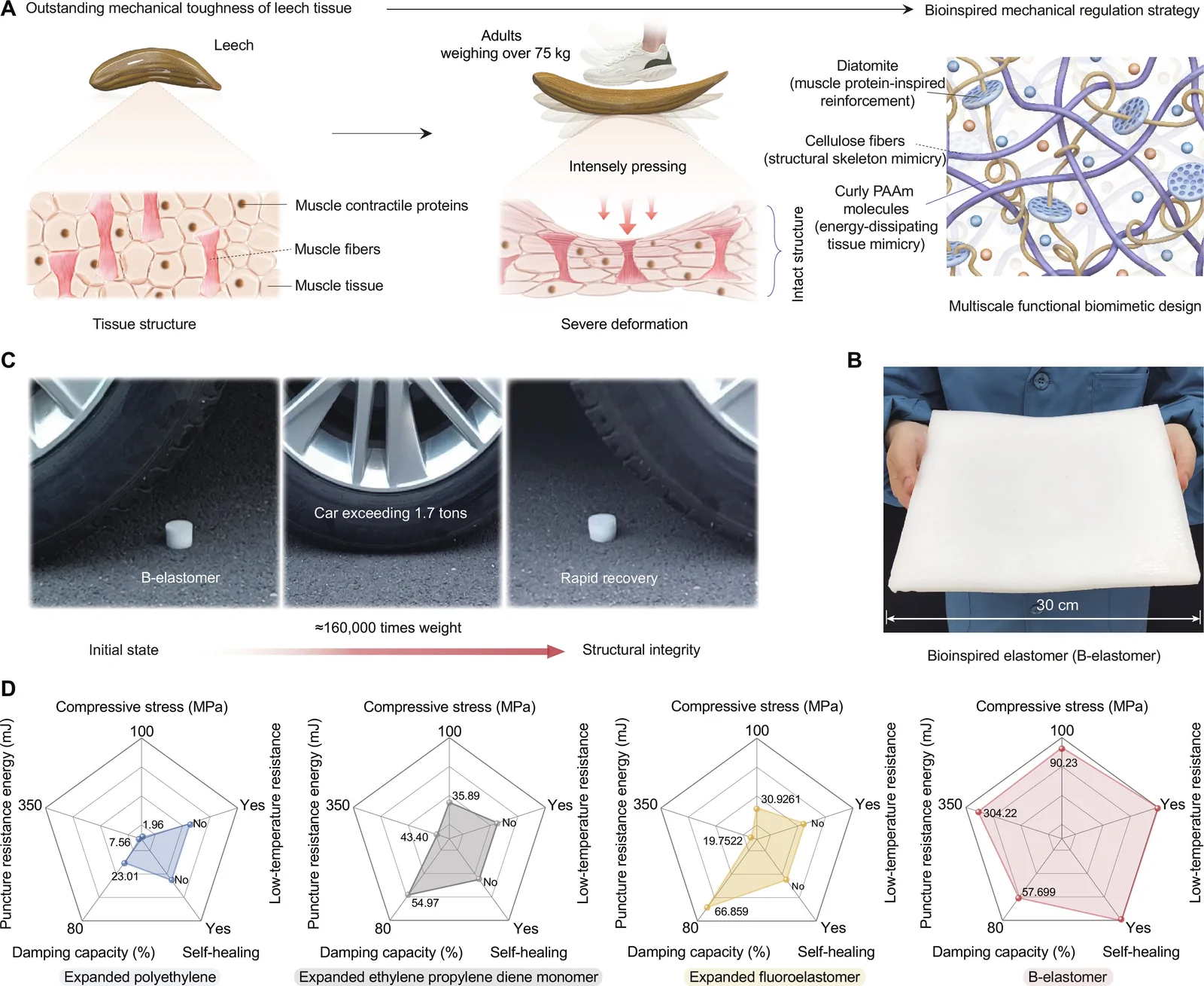
Design strategy and characterization of B-elastomer. (A) Schematic of the preparation of leech-inspired B-elastomer. (B) Optical images showing the large-scale preparation of B-elastomer. (C) Investigating the compressive resistance and shape recovery of B-elastomer. (D) Comparison of key performance between B-elastomer and commercial elastomers.

Inspired by the synergistic mechanical functions of leech tissues, we fabricate a bioinspired cellulosic elastomer (B-elastomer) through ethanol-induced molecular rearrangement and densification-driven reconstruction of the cellulose, nanodiatomite, and polyacrylamide (PAAm) network. In B-elastomer, cellulose chains and nanodiatomite form a structural framework that facilitates stress transfer and energy dissipation, while the PAAm network provides deformability and elastic toughness. Benefiting from this leech-inspired architecture, B-elastomer achieves a compressive strength of 90.23 MPa at 95% strain and a compressive modulus of about 400 MPa. The elastomer also exhibits outstanding resistance to static puncture and dynamic impact, together with self-healing capability and retained toughness at temperatures as low as −196 °C. This bioinspired design enables scalable production of high-performance elastomers and offers attractive economic viability.

## Results

### Fabrication and performance of B-elastomer

By leveraging the distinct conformational responses of PAAm molecular chains and cellulose chains under ethanol stimulation, we constructed a bioinspired B-elastomer. During the process, cellulose chains undergo limited contraction and progressively assemble into a rigid, interconnected framework, while PAAm molecules experience pronounced rearrangement and gradually curl under ethanol induction, forming a compliant matrix that interpenetrates the rigid cellulose framework [29]. The incorporation of nanoscale diatomite further reinforces this multiscale structure, producing a load-bearing architecture capable of efficiently distributing stress. As shown in Fig. 1B, a white B-elastomer, measuring up to 30 cm in size, is displayed.

In B-elastomer, cellulose establishes a continuous reinforcing framework, nanodiatomite acts as rigid reinforcing domains, and the PAAm network provides a flexible matrix for deformation accommodation and energy dissipation (Fig. 1A). The functional organization resembles the division of roles in biological tissues, where different structural components cooperatively regulate mechanical responses under external loading. The abundant hydrogen-bonding interactions originate from silanol groups in nanodiatomite, hydroxyl groups enriched in cellulose chains, and amide groups in PAAm molecules. Coupled with the interpenetration and entanglement of PAAm with the hybrid network, these dynamic interactions increase the effective physical cross-linking density, endowing B-elastomer with outstanding mechanical properties. As shown in Fig. 1C, B-elastomer can support a car weighing more than 1.7 tons, approximately 160,000 times its own weight, and rapidly recover its original shape after the load is removed. Moreover, by simply applying a small amount of water to the damaged region to induce bioinspired network reorganization, B-elastomer exhibits remarkable room-temperature self-healing capability. Compared with 3 commercial elastomers, namely, expanded polyurethane, expanded ethylene propylene diene monomer, and expanded fluoroelastomer, B-elastomer demonstrates superior mechanical performance as well as self-healing ability (Fig. 1D).

### Ethanol-induced structural evolution of B-elastomer

B-elastomer before ethanol stimulation was designated as B-gel. As shown in Fig. 2A, B-gel consists of a hydrated network composed of PAAm, cellulose, and nanodiatomite. Upon ethanol stimulation, water-to-ethanol solvent exchange disrupts the original hydration environment and water-mediated interactions within the network, inducing pronounced conformational rearrangement of PAAm chains and subsequent network reconstruction. The presence of the cellulose framework and nanodiatomite influences this rearrangement, promoting the formation of a more compact B-elastomer network.

**Fig. 2.**
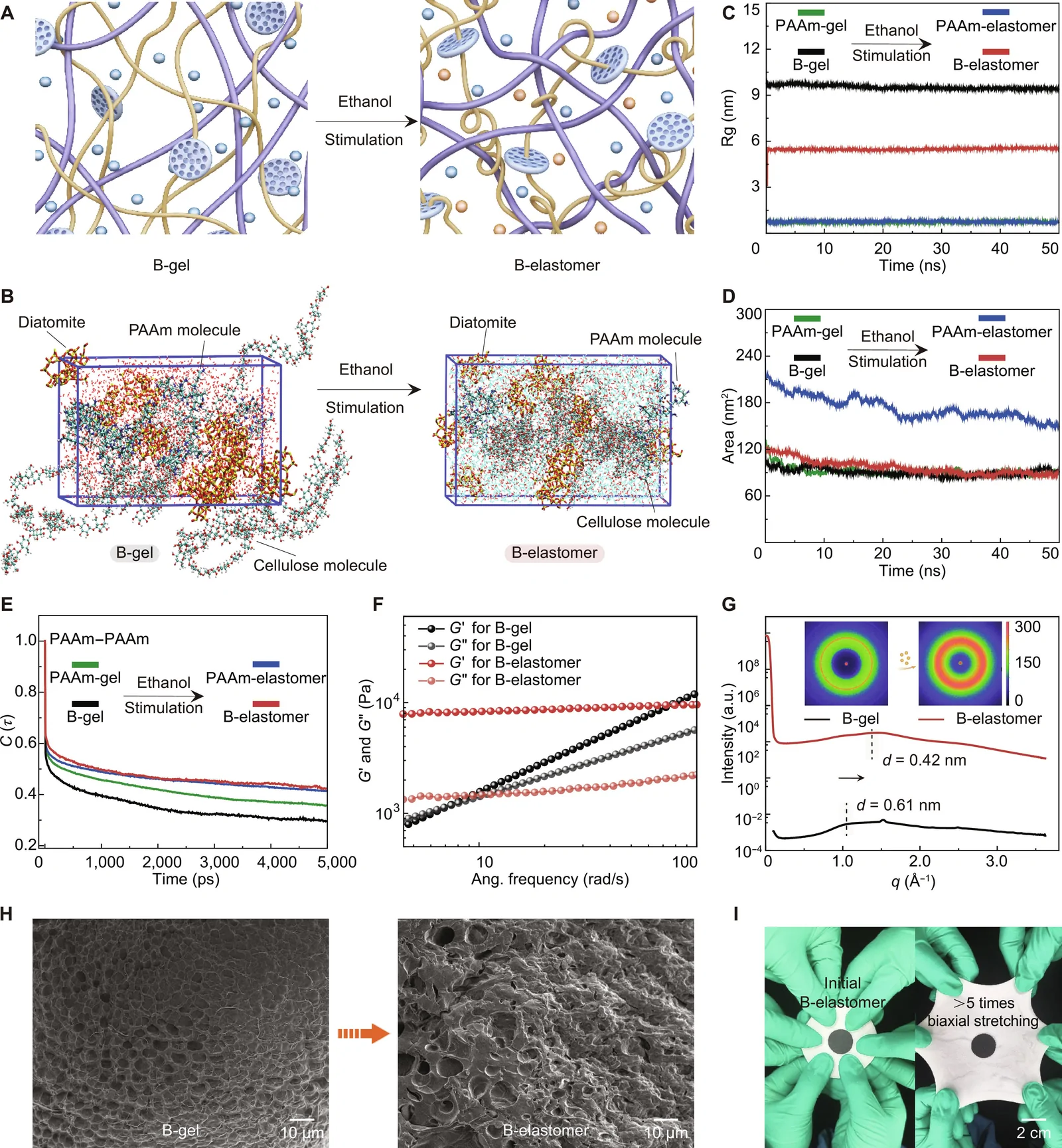
Construction mechanisms, molecular dynamics simulations, and morphological structure of B-elastomer. (A) Schematic diagram illustrating the construction of bioinspired architecture within B-elastomer. Blue spheres represent water molecules, and orange spheres represent ethanol molecules. (B) Representative molecular dynamics snapshots of B-gel and B-elastomer. (C) Radius of gyration (Rg) in PAAm-gel, PAAm-elastomer, B-gel, and B-elastomer. PAAm, polyacrylamide. (D) Solvent-accessible surface area of PAAm molecules in PAAm-gel, PAAm-elastomer, B-gel, and B-elastomer. (E) H-bond lifetime autocorrelation curves of PAAm–PAAm in PAAm-gel, PAAm-elastomer, B-gel, and B-elastomer. (F) Comparison of storage moduli (*G*′) and loss moduli (*G*″) between B-gel and B-elastomer. (G) The wide-angle x-ray scattering (WAXS) curves of B-gel and B-elastomer (inset: the WAXS 2-dimensional [2D] patterns of B-gel and B-elastomer). (H) Scanning electron microscopy (SEM) images of B-gel and B-elastomer. (I) Optical images of B-elastomer showing biaxial stretching behavior.

The structural evolution of B-elastomer was investigated by combining molecular, nanoscale, and microscale characterization. Molecular dynamics simulations captured the structural evolution at the molecular level, revealing the transition from the relatively extended configuration of B-gel to the more compact and reorganized network of B-elastomer (Fig. 2B). Consistent with this structural transition, the radius of gyration (Rg) of PAAm decreased after ethanol stimulation, reflecting conformational rearrangement of the PAAm chains during network reconstruction (Fig. 2C). In B-elastomer, Rg decreased from 9.42 to 5.54 nm, revealing a pronounced change in the PAAm molecular chain conformation (Fig. 2C and Fig. S1A). Compared with the neat PAAm system (including PAAm-gel and PAAm-elastomer), the conformational evolution of PAAm within the cellulose/diatomite-containing network (including B-gel and B-elastomer) was more constrained, suggesting that the rigid cellulose framework and nanodiatomite components restricted excessive molecular rearrangement during ethanol-induced reconstruction.

PAAm-elastomer exhibited a substantially higher solvent-accessible surface area than B-elastomer, whereas B-gel and B-elastomer maintained relatively similar values (Fig. 2D and Fig. S1B). The incorporation of cellulose and nanodiatomite suppresses the increase in molecular accessibility observed in the neat PAAm system and constrains PAAm rearrangement within the composite network. Meanwhile, B-elastomer showed a slower hydrogen-bond autocorrelation decay than B-gel, indicating more persistent intermolecular associations after ethanol stimulation (Fig. 2E). Thus, the molecular dynamics simulation results suggest that ethanol stimulation alters the original hydrated environment and induces PAAm conformational rearrangement, accompanied by more persistent intermolecular associations in the reconstructed network.

Rheological characterization revealed that B-elastomer exhibited higher storage modulus (*G*′) and loss modulus (*G*″) than B-gel across the measured frequency range (Fig. 2F). *G*′ exceeded 10^4^ Pa, and *G*″ was significantly increased, indicating enhanced network stiffness and improved energy dissipation for B-elastomer. Additionally, the larger separation between the *G*′ and *G*″ curves of B-elastomer highlighted more robust viscoelastic behavior, indicating that the network could adapt under stress and effectively alleviate local strain concentration. Compared with the PAAm-elastomer lacking bioinspired architecture, B-elastomer showed a lower tan *δ* value (Fig. S2), reflecting a more elastic-dominated network response.

Wide-angle x-ray scattering (WAXS) analysis provided insight into nanoscale structure evolution. B-gel displayed isotropic halos characteristic of amorphous and randomly oriented chains, while B-elastomer exhibited distinct scattering rings (Fig. 2G, inset, and Fig. S3A). The WAXS profiles showed a shift of the correlation peak from *q* = 0.0162 Å^−1^ to *q* = 0.0283 Å^−1^ (Fig. 2G and Fig. S3B), corresponding to a reduced characteristic intermolecular/interdomain distance after ethanol stimulation, indicating a more compact network organization within B-elastomer.

Small-angle x-ray scattering patterns confirmed similar trends. Figure S4 exhibits that B-gel showed weak isotropic scattering, whereas B-elastomer exhibited much brighter scattering centers. The low-*q* scattering intensity of B-elastomer was markedly enhanced compared with that of B-gel, reflecting the formation of PAAm-rich microdomains with abundant physical cross-links and entanglements in B-elastomer (Fig. S5). This densified PAAm molecular network enhanced the structural connectivity and impact toughness of B-elastomer, while the embedded cellulose framework and nanodiatomite promoted stress redistribution and improved structural strength.

Scanning electron microscopy (SEM) images revealed clear differences in the microstructures of B-gel and B-elastomer (Fig. 2H). B-gel exhibited a loosely connected and porous morphological structure. In contrast, B-elastomer displayed a dense and interconnected network structure. Higher-magnification SEM images further confirmed this structural densification after ethanol stimulation (Fig. S6). Additionally, the ^1^H solid-state nuclear magnetic resonance spectra provided further information on changes in the local proton environments during ethanol-induced reconstruction. As shown in Fig. S7A, B-gel exhibited a dominant broad signal at approximately 4.5 to 4.7 ppm, together with weaker signals at 1.45 and 1.28 ppm. The broad signal in this region likely contains overlapping contributions from multiple proton environments, including hydroxyl-containing components and residual bound water, making an unambiguous assignment of individual contributions difficult. After ethanol stimulation, B-elastomer exhibited distinct signals at 4.58, 3.74, 3.05, and 0.63 ppm, indicating a pronounced redistribution of local proton environments. Notably, PAAm-elastomer exhibited highly similar signals at 4.58, 3.82, 3.09, and 0.65 ppm (Fig. S7B), indicating that the major spectral evolution of B-elastomer is predominantly associated with ethanol-induced changes in the local molecular environment of PAAm. These results are consistent with PAAm rearrangement during ethanol-induced network reconstruction. The resulting compact and interconnected network is consistent with the robust mechanical behavior of B-elastomer under large deformation. As shown in Fig. S8, B-elastomer withstood severe mechanical deformations, including twisting and localized compression under a 2.5-kg load. Furthermore, B-elastomer demonstrated remarkable biaxial stretchability, extending over 5 times its original length while maintaining structural integrity (Fig. 2I).

### Strong mechanical properties

The compressive stress–strain curves of B-elastomer exhibited a staged transition from an initial compliant regime to rapid strain stiffening at large deformations (Fig. 3A). B-elastomer achieved a compressive strength of 90.23 MPa and a compressive modulus of 399.99 MPa, representing an ~470% improvement over PAAm-elastomer (Fig. 3B). Cyclic compression loading–unloading experiments were conducted on B-elastomer to investigate its energy dissipation and damping evolution at different strain levels. As shown in Fig. 3C, the damping of B-elastomer increased with compressive strain, reaching 20.90 MJ m^−3^ at 90% strain, indicating substantial energy dissipation (Fig. S9). The damping capacity of B-elastomer remained relatively stable during 50 repeated high-strain compression cycles (Fig. 3D and E and Fig. S10), with no appreciable attenuation of energy-dissipation capability, which demonstrates that the ethanol-induced reconstructed network can withstand repeated mechanical deformation while maintaining stable energy-dissipation performance. Compared with previously reported materials and representative commercial elastomers, B-elastomer still demonstrated superior compressive stress and modulus at the same strain (Fig. 3F and Figs. S11 and S12) [30–38]. These results indicate that the leech-inspired architecture endows B-elastomer with sustained energy dissipation, enabling resistance to cyclic fatigue under external forces and ensuring both structural integrity and rapid recovery.

**Fig. 3.**
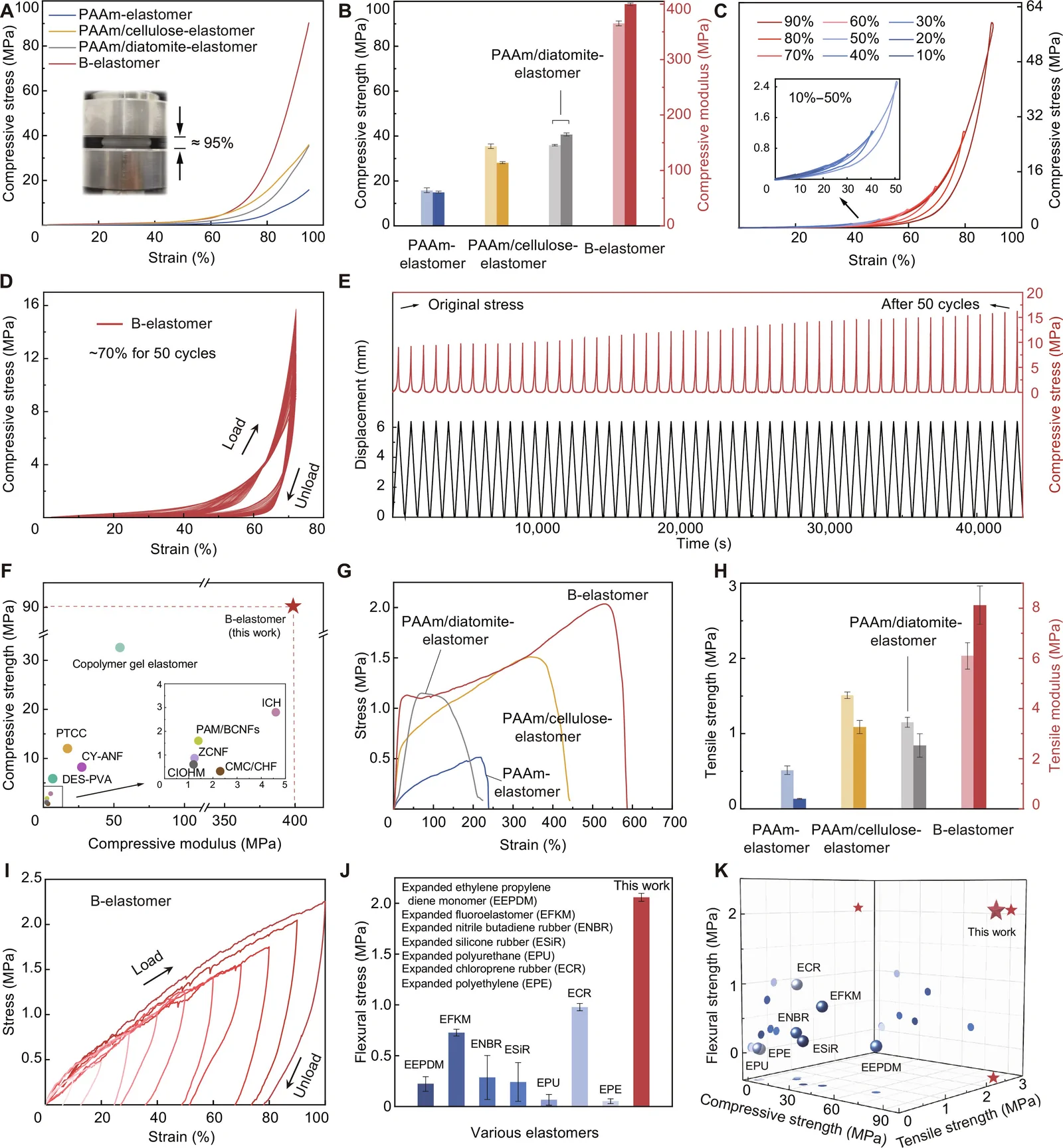
Robust mechanical performance of B-elastomer. (A) Compressive stress–strain curves and (B) comparison of the compressive strengths and moduli of PAAm-elastomer, PAAm/cellulose-elastomer, PAAm/diatomite-elastomer, and B-elastomer. Insets show the digital images of B-elastomer with 95% compression. PAAm, polyacrylamide. (C) Sequential loading–unloading compressive cycles of B-elastomer with incremental strains from 10% to 90%. (D) Cyclic compressive stress–strain curves of B-elastomer. (E) Investigating the cycling compressive performance of B-elastomer over 50 cycles. (F) Comparison of compression mechanical performance between B-elastomer and previously reported elastomers. (G) Tensile stress–strain curves and (H) comparison of the tensile strengths and moduli of PAAm-elastomer, PAAm/cellulose-elastomer, PAAm/diatomite-elastomer, and B-elastomer. (I) Sequential loading–unloading tensile cycles of B-elastomer. (J) Flexural strength of several commercial elastomers and B-elastomer. (K) Comparison of compression strengths, tensile strengths, and flexural strengths between B-elastomer and several commercial elastomers. Data in (B), (H), and (J) are expressed as mean value ± standard deviation (*n* = 3).

As shown in Fig. 3G, B-elastomer exhibited a tensile strength of 2.03 MPa, an elongation at break of nearly 600%, and a tensile modulus of 8.11 MPa (Fig. 3H), which were higher than those of elastomers lacking bioinspired structures (Figs. S13 and S14). During stepwise tensile loading–unloading cycles over a strain range of 10% to 100% (Fig. 3I), B-elastomer displayed pronounced hysteresis at each deformation level, confirming efficient energy dissipation. Three-point bending tests further showed that B-elastomer possessed a high flexural strength of 2.05 MPa (Fig. 3J), demonstrating excellent structural stability under substantial loading. As summarized in Fig. 3K, B-elastomer outperforms these commercial elastomers in compressive strength, tensile deformability, and strain-dependent energy dissipation.

### Finite element analysis and puncture resistance

Finite element analysis (FEA) was conducted to investigate the stress distribution behavior in B-elastomer under compression and tension states. As shown in Fig. 4A, B-elastomer underwent a compression cycle in which the strain increased from 0% to 50% and then to 95%, followed by recovery to its initial state. From observing the compressive stress distribution nephograms obtained from FEA, the cellulose framework within B-elastomer, compared to PAAm-elastomer (Fig. S15), bore the primary load, while the dispersed diatomite interrupted stress concentration and redistributed stresses into the surrounding PAAm molecular network (Fig. 4B). This bioinspired architecture enables B-elastomer to undergo a transition during deformation from an initially flexible state to strain hardening and then to a dense protective state, endowing it with outstanding compressive resistance as well as high mechanical strength (Fig. 4C and Fig. S16). During tensile deformation, B-elastomer exhibits a tensile strain exceeding 5 times its initial value (Fig. 4D), while the stress distribution remains uniform along the loading direction, with no evidence of stress concentration (Fig. 4E). This behavior is attributed to the cellulose framework, which facilitates rapid axial load transfer (Fig. S17). Compared with single-component elastomers, B-elastomer shows superior tensile performance and structural strength at the same tensile strain (Fig. 4F and Fig. S18).

**Fig. 4.**
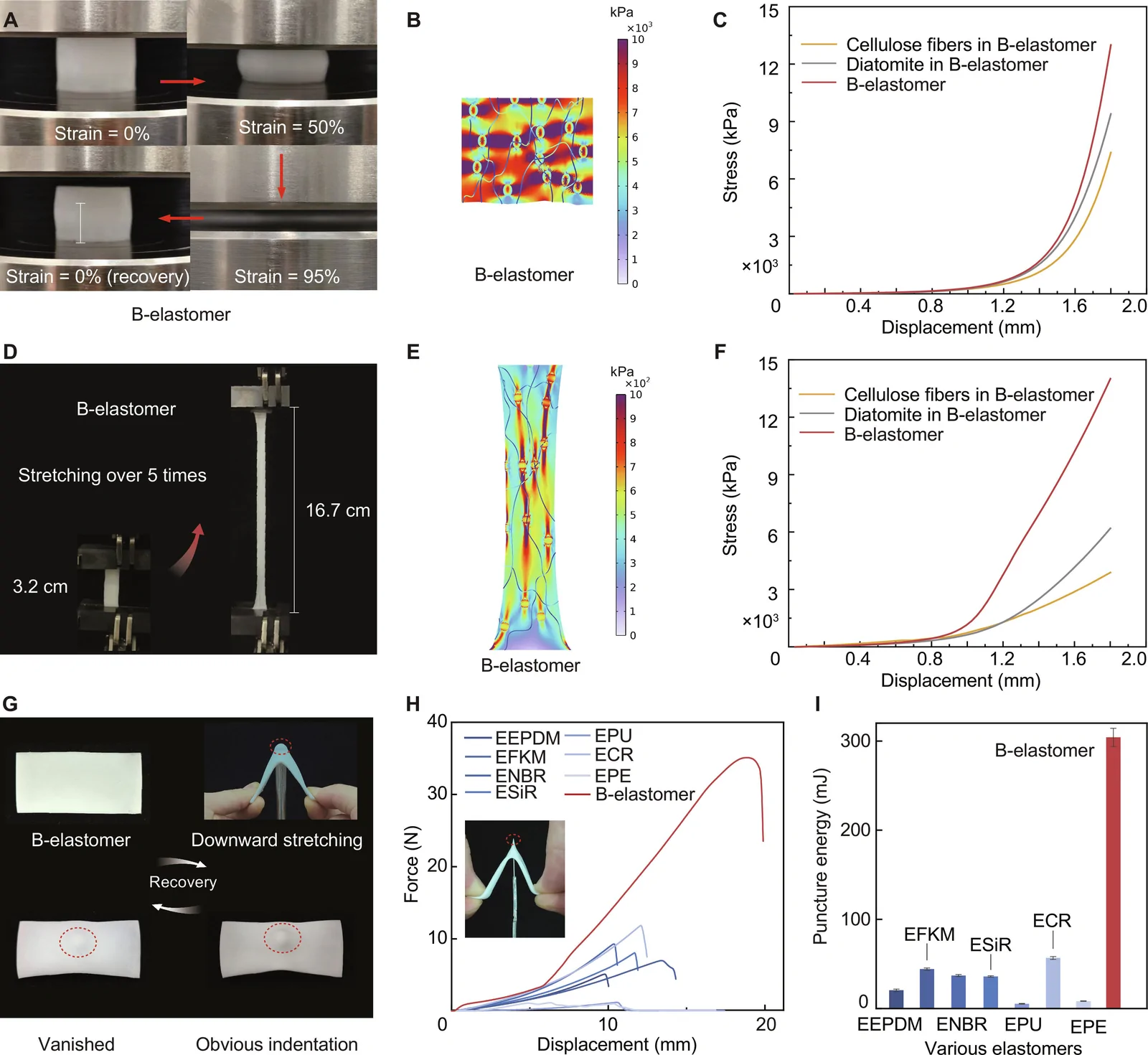
Finite element analysis (FEA) and compelling puncture resistance of B-elastomer. (A) Optical images of B-elastomer undergoing 95% compression and subsequent complete recovery. (B) Compressive stress distribution nephograms of B-elastomer obtained from FEA. (C) Compressive stress–displacement curves obtained from FEA of cellulose chains in B-elastomer, diatomite in B-elastomer, and B-elastomer. (D) Optical images of B-elastomer showing its attractive mechanical stretchability. (E) Tensile stress distribution nephograms of B-elastomer obtained from FEA. (F) Tensile stress–displacement curves of cellulose chains in B-elastomer, diatomite in B-elastomer, and B-elastomer obtained from FEA. (G) Optical images of B-elastomer remaining intact after static puncture and subsequent complete recovery. (H) Puncture curves and (I) puncture energy of several commercial elastomers and B-elastomer. The inset shows a digital image of B-elastomer with static puncture. The data in (I) are expressed as mean value ± standard deviation (*n* = 3).

In addition, static puncture tests under concentrated loading demonstrated that B-elastomer can withstand deep indentation without catastrophic fracture and fully recover its original shape after the external force is removed (Fig. 4G), validating the FEA of the dissipation mechanism in B-elastomer. Compared with commercial elastomers and previously reported elastomers, B-elastomer exhibits superior puncture resistance (Fig. 4H and Fig. S19). Even when subjected to an external force exceeding 30 N applied by the tip of a surgical scalpel (inset in Fig. 4H), B-elastomer remained structurally intact and undamaged with a puncture-resistant energy of 304.22 mJ (Fig. 4I), which is more than 3 times that of commercial expanded chloroprene rubber (ECR) elastomers.

### Impact-resistant, self-healing, and anti-low-temperature for robust protection

The dynamic impact performance of B-elastomer was subsequently examined using a split-Hopkinson pressure bar system in Fig. 5A. As shown in Fig. 5B, the impact stress–strain curves demonstrated that B-elastomer had a pronounced strain-rate-strengthening behavior, with the peak stress increasing from 19.43 MPa at 5 m s^−1^ to 77.90 MPa at 30 m s^−1^. The volumetric energy absorption, impact toughness, and energy-dissipation factor of B-elastomer also increased with increasing impact velocity, reaching 54 MJ m^−3^, 28.62 MJ m^−3^, and 0.295 at 30 m s^−1^, respectively (Fig. 5C and D and Fig. S20). The reliability of dynamic measurements was supported by the incident, reflected, and transmitted strain signals of B-elastomer shown in Fig. S21. These signals confirmed that a dynamic stress equilibrium was established in B-elastomer during loading, enabling it to exhibit impressive structural integrity under high-speed impact and to rapidly recover its original shape after external force was removed, without impact-induced cracking.

**Fig. 5.**
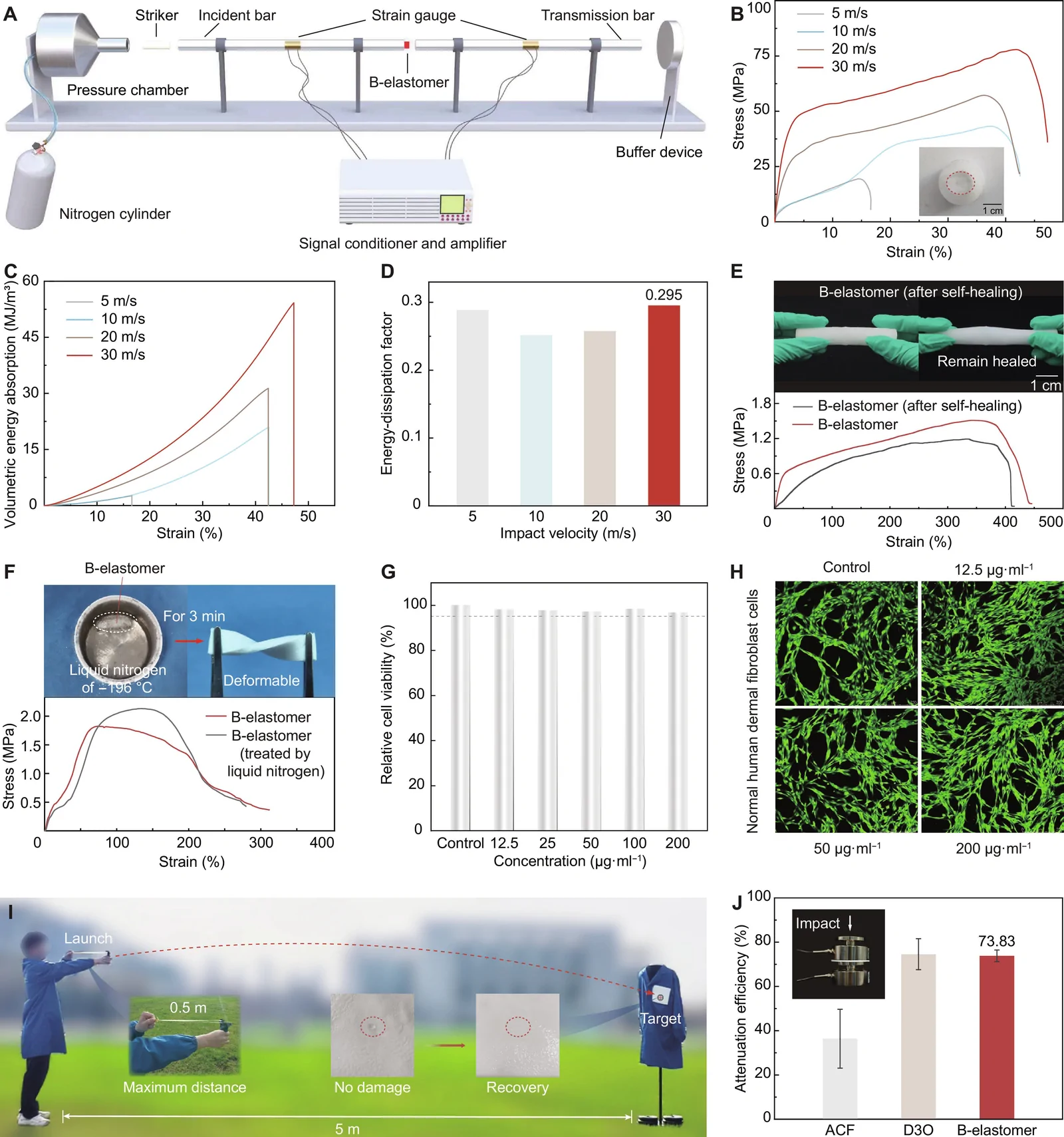
Impact resistance, self-healing properties, low-temperature resistance, and noncytotoxicity of B-elastomer. (A) Schematic illustration of the split-Hopkinson pressure bar (SHPB) setup for evaluating the impact resistance of B-elastomer. (B) Mechanical impact stress–strain curves, (C) volumetric energy absorption, and (D) energy-dissipation factor of B-elastomer at different incident speeds. (E) Compressive stress–strain curves of B-elastomer before and after self-healing, with an inset showing that the self-healed B-elastomer can still be compressed to 95% strain and fully recover. (F) Tensile stress–strain curves of B-elastomer before and after liquid nitrogen treatment. The insets demonstrate the good deformable behavior of B-elastomer in liquid nitrogen at −196 °C for 3 min. (G) Investigating the cell viability of B-elastomer in different cell suspension concentrations. (H) Live staining confocal images of normal human dermal fibroblasts cells co-cultured with different concentrations of B-elastomer for 24 h. (I) Optical images of B-elastomer serving as a wearable protective layer under impact from a steel ball launched from 5 m. (J) Attenuation efficiency of artificial cartilage foam (ACF), D3O, and B-elastomer. The inset shows a digital image of B-elastomer with impact test. The data in (J) are expressed as mean value ± standard deviation (*n* = 3).

Beyond its excellent impact-resistant properties, B-elastomer also exhibits self-healing capabilities. At room temperature, water was applied to the interface between 2 B-elastomer sheets to activate the stretching of bioinspired architecture and hydrogen-bond interactions, followed by ethanol stimulation to reconstruct an interface-spanning, densified network (Fig. S22). The resulting self-healing B-elastomer exhibited tensile and compressive performance comparable to that of the original B-elastomer (Fig. 5E and Fig. S23) and strong mechanical peel behavior (Fig. S24, peel force exceeding 39 N mm^−1^). This simple and easy-to-control self-healing behavior enables B-elastomer to be reintegrated, reorganized, and recycled after damage and decommissioning.

In addition, B-elastomer demonstrated excellent low-temperature resistance. As shown in Fig. 5F, after continuous immersion in liquid nitrogen at −196 °C for 3 min, B-elastomer still retained bendable deformability and mechanical tensile stress–strain behavior comparable to that in its initial state. In addition, biocompatibility tests showed that across a range of B-elastomer extract concentrations, cell viability remained above 90% (Fig. 5G). The cells also maintained normal morphology and showed no apparent differences from the controls (Fig. 5H), indicating the low cytotoxicity of B-elastomer.

As a wearable protective layer, B-elastomer effectively mitigates the impact of a steel ball launched from 5 m, with rapid shape recovery and preserved structural integrity (Fig. 5I). Compared with representative impact-resistant materials, B-elastomer achieves an attenuation efficiency of 73.83%, approaching that of D3O (Fig. 5J). Owing to its outstanding mechanical robustness, self-healing capability, low-temperature resistance, and biocompatibility, B-elastomer holds strong potential as a soft protective material for human bodies, precision instruments, and intelligent robots.

### Scalable manufacturing process and economic feasibility

The production of elastomers on a large scale is often limited by the absence of continuous, energy-efficient fabrication strategies. To address the limitation, a continuous and scalable manufacturing process for B-elastomer was developed (Fig. 6A). In the process, the precursor solution was continuously mixed and delivered onto a heated conveyor belt, where thermal polymerization induced the formation of B-gel while enabling water vapor removal. Then, the B-gel underwent solvent-induced network densification upon immersion in an ethanol bath to fabricate B-elastomer. Meanwhile, a closed-loop system facilitated recovery and reuse of both water and ethanol, minimizing solvent consumption and improving process sustainability. Additionally, B-elastomer could be easily processed into customizable sheets via die-cutting. As shown in Fig. 6B, a large-scale B-elastomer exceeding 100 cm in length, 25 cm in width, and 5 mm in thickness was successfully fabricated (Fig. 6C).

**Fig. 6.**
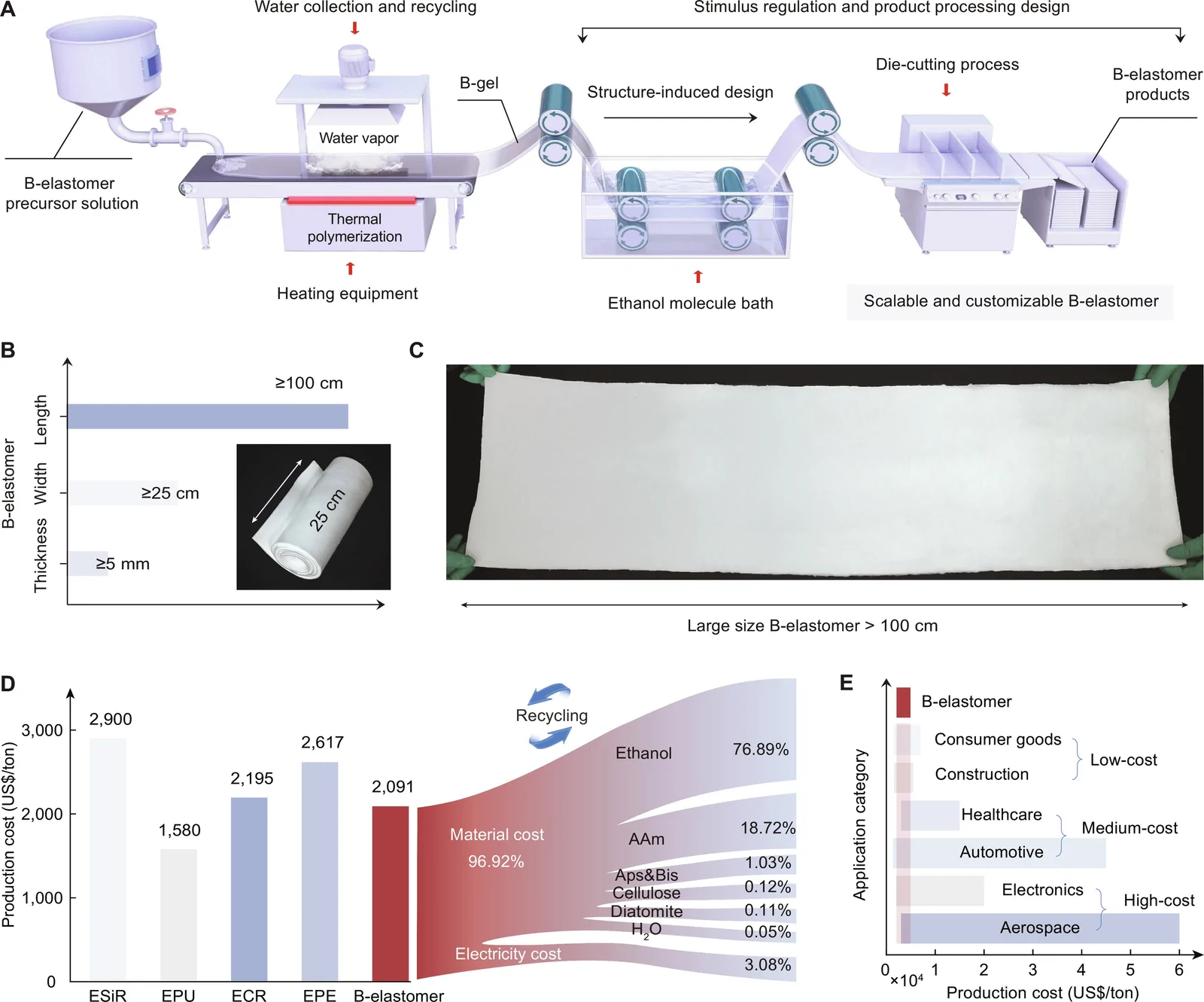
Manufacturing process and economic feasibility of B-elastomer. (A) Schematic diagram of the industrial manufacturing process for B-elastomer. (B) Dimensions and (C) optical images of a large-scale piece of B-elastomer. (D) Comparison of the production costs of B-elastomer and several commercial elastomers, namely, expanded silicone rubber (ESiR), expanded polyurethane (EPU), expanded chloroprene rubber (ECR), and expanded polyethylene (EPE). (E) Cost competitiveness of B-elastomer across targeted application fields.

Beyond scalable fabrication, the economic viability of B-elastomer was evaluated by a technoeconomic analysis in comparison with several commercial elastomers, including expanded silicone rubber, expanded polyurethane, ECR, and expanded polyethylene (Fig. 6D and Tables S1 and S2). The production cost of B-elastomer was approximately US$2,091 per ton, which is comparable to that of ECR. Ethanol dominated the overall cost, accounting for 76.89% of the total raw material cost. Electricity and water contributed less than 3.2%, indicating relatively low energy consumption. Notably, improving solvent recovery efficiency could substantially reduce the overall cost, providing further economic optimization.

Depending on application requirements, elastomers were categorized into low-, mid-, and high-cost systems (Fig. 6E). Low-cost elastomers prioritize manufacturability and are typically served as consumer and construction products, whereas mid-cost elastomers balance mechanical reliability and environmental resistance for medical and automotive applications. High-cost elastomers are designed for demanding applications, such as medical-grade, smart electronics, and aerospace materials, where performance and reliability surpass cost considerations. Our B-elastomer fell within the mid- to low-cost range while providing high mechanical performance and multifunctionality, positioning it as a promising candidate for next-generation protective applications.

## Conclusion

Inspired by the leech’s stress dissipation mechanisms, we developed a B-elastomer by integrating cellulose and nanodiatomite with the PAAm molecular network through an ethanol-induced process. Ethanol regulated PAAm chain conformations, facilitating reversible network formation and regional densification. The cellulose molecules served as the primary load-bearing framework, while dispersed nanodiatomite within the PAAm network further reinforced the network architecture. This cooperative bioinspired architecture imparted B-elastomer with a synergistic combination of mechanical strength, toughness, and resilience. For example, B-elastomer exhibited a compressive strength of over 90 MPa at 95% strain and a tensile strength of over 2 MPa with an elongation approaching 600% and retained high stability under cyclic loading. It also showed pronounced resistance to puncture and impact, along with self-recovery, while preserving mechanical integrity at temperatures as low as −196 °C.

In addition, B-elastomer was amenable to continuous and roll-to-roll processing design, allowing meter-scale fabrication with reduced energy consumption. The scalable and sustainable approach enabled B-elastomer to deliver robust mechanical performance within a low- to mid-cost range, proving that high-performance elastomers can be achieved through structural engineering. Looking forward, tuning the degree of structural densification and evaluating long-term fatigue and reliability are critical for the practical deployment of these materials. This work established a versatile framework for high-performance, sustainable elastomers and holds promises for protective gears, impact-resistant systems, and structural damping applications.

## Materials and Methods

### Materials

Poplar flour (60 mesh) was obtained from Lingshou County Ningbo Mineral Products Co., Ltd. (Shijiazhuang, Hebei, China). Sodium chlorite (NaClO_2_, ≥80%), glacial acetic acid (≥99.5%), potassium hydroxide (KOH, AR, ≥85%), acrylamide (AM, AR, ≥99%), ammonium persulfate (Primor Trace, ≥99.99% metal basis), and *N*,*N*′-methylenebis(acrylamide) (Bis, CP, ≥97%) were purchased from Shanghai Aladdin Biochemical Technology Co., Ltd. (Shanghai, China). Diatomite (SiO_2_ ≥ 85% and D50 = 7 to 20 μm) was purchased from Jilin Yuantong Mining Co., Ltd. (Linjiang, Jilin, China). Anhydrous ethanol (AR, ≥99.7%) was purchased from Tianjin Fuyu Fine Chemical Co., Ltd. (Tianjin, China).

### Preparation of cellulose

Cellulose was extracted from poplar flour through sequential delignification and hemicellulose removal, followed by ultrasonic fibrillation. Briefly, poplar flour (30 g, 60 mesh) was dispersed in distilled water (1,500 g), followed by the addition of sodium chlorite (15 g) and glacial acetic acid (3 ml). The mixture was sealed and maintained in a water bath at 75 °C for 1 h with stirring every 30 min. Subsequently, sodium chlorite (15 g) and glacial acetic acid (3 ml) were added again, and this treatment was repeated 5 times for delignification. After the reaction, the mixture was cooled to room temperature and washed repeatedly with distilled water. The product was then collected by vacuum filtration and washed with distilled water until neutral pH was reached to obtain holocellulose.

The obtained holocellulose was subsequently treated with potassium hydroxide to remove hemicellulose. Potassium hydroxide (40 g) was dissolved in a small amount of distilled water, followed by the addition of holocellulose and distilled water to give a total mass of 800 g. The mixture was maintained in a water bath at 90 °C for 2 h. After treatment, the product was collected by vacuum filtration and repeatedly washed with distilled water until neutral pH was reached.

To further remove residual lignin and hemicellulose, delignification and alkaline treatments were performed again. The partially purified cellulose was dispersed in distilled water (1,500 g), followed by the addition of sodium chlorite (15 g) and glacial acetic acid (3 ml). The mixture was maintained in a water bath at 75 °C for 2 h with stirring every 30 min. After 1 h, an additional 15 g of sodium chlorite and 3 ml of glacial acetic acid were added. After the reaction, the product was cooled to room temperature, collected by vacuum filtration, and repeatedly washed with distilled water until neutral pH was reached. Subsequently, potassium hydroxide (40 g) was dissolved in a small amount of distilled water, followed by the addition of the obtained cellulose and distilled water to give a total mass of 800 g. The mixture was maintained in a water bath at 90 °C for 1 h. The product was then collected by vacuum filtration and repeatedly washed with distilled water until neutral pH was reached to obtain purified cellulose.

Finally, the purified cellulose was dispersed in distilled water at a concentration of 2 wt.% and subjected to ultrasonic fibrillation using an ultrasonic processor (SCIENTZ-1500F, Ningbo Scientz Biotechnology Co., Ltd., China) at a frequency of 20 kHz. Ultrasonication was performed in a pulsed mode of 3 s on and 2 s off. The cellulose dispersion was sequentially sonicated at 600 W for 6 min, 900 W for 10 min, and 450 W for 6 min. An ice-water bath was used throughout the ultrasonication process to minimize the temperature increase. After ultrasonication, the obtained cellulose dispersion was stored under refrigeration until further use. The obtained cellulose exhibited a fibrillar morphology with nanoscale fibrillar structures (Fig. S25) and an average degree of polymerization of approximately 1,500.

### Pretreatment of diatomite

Nanodiatomite (SiO_2_ ≥ 85% and D50 = 7 to 20 μm) was pretreated before use to remove soluble impurities. Here, nanodiatomite refers to diatomite with abundant nanoscale pores distributed on its microscale skeleton, rather than nanosized diatomite particles, as revealed by SEM observations (Fig. S26). Briefly, the nanodiatomite was immersed in anhydrous ethanol and ultrasonicated in an ultrasonic cleaner at 150 W for 3 min. The resulting suspension was filtered using qualitative filter paper to remove the ethanol containing soluble impurities. The collected nanodiatomite was subsequently dried in an oven at 80 °C for 1 h and collected for further use.

### Fabrication of B-gel

The prepared 2 wt.% cellulose dispersion was used directly, while diatomite was ultrasonically dispersed in distilled water to prepare a 2 wt.% diatomite suspension. The AM monomer solution was prepared following reported methods [39–41]. Briefly, 80 g of AM was dissolved in 500 g of water and stirred for 30 min, followed by the addition of 0.4 g of ammonium persulfate as the initiator and 0.08 g of Bis as the cross-linker. The solution was further stirred for 20 min to ensure complete dissolution. The AM monomer solution, cellulose dispersion, and diatomite suspension were then mixed at a weight ratio of 24:5:5 and stirred at a high speed on an orbital shaker for 1 h. The resulting suspension was quickly transferred into molds and degassed in a vacuum oven at 20 °C under a vacuum of −0.04 MPa for 10 min to remove air bubbles. Subsequently, the molds were placed in an oven at atmospheric pressure and 65 °C for 2 h to complete the polymerization, obtaining the B-gel.

The concentrations of the cellulose dispersion and diatomite suspension were selected based on preliminary formulation screening while maintaining the composition of the AM monomer solution and the weight ratio of AM monomer solution/cellulose dispersion/diatomite suspension at 24:5:5. Cellulose dispersion/diatomite suspension concentrations of 1/1, 1/2, 2/1, 2/2, 3/3, and 5/5 wt.% were investigated. The formulations were evaluated based on suspension stability and polymerization behavior, and the successfully polymerized samples were subsequently subjected to the same ethanol treatment to obtain the corresponding elastomers for tensile testing. Based on these results (Figs. S27 and S28), 2 wt.% cellulose dispersion and 2 wt.% diatomite suspension were selected for the preparation of B-elastomer.

### Fabrication of B-elastomer

B-elastomer was obtained by immersing B-gel in an 80% (v/v) ethanol aqueous solution at room temperature for 12 h, until it turned macroscopically white and mechanically resilient. The successfully polymerized samples obtained during formulation screening were subjected to the same ethanol treatment before the tensile test. The ethanol concentration and immersion time were optimized based on the results shown in Figs. S29 and S30.

### Fabrication of PAAm-elastomer

The prepared AM monomer solution was transferred into molds and degassed in a vacuum oven at 20 °C under a vacuum of −0.04 MPa for 10 min to remove air bubbles. The molds were subsequently placed in an oven at atmospheric pressure and 65 °C for 2 h to complete the polymerization, obtaining PAAm-gel. PAAm-gel was then immersed in an 80% (v/v) ethanol aqueous solution at room temperature for 12 h to obtain PAAm-elastomer.

### Fabrication of PAAm/cellulose-elastomer

The prepared AM monomer solution and 2 wt.% cellulose dispersion were mixed at a weight ratio of 12:5 and stirred at a high speed on an orbital shaker for 1 h. The resulting suspension was transferred into molds and degassed in a vacuum oven at 20 °C under a vacuum of −0.04 MPa for 10 min to remove air bubbles. The molds were subsequently placed in an oven at atmospheric pressure and 65 °C for 2 h to complete the polymerization, obtaining PAAm/cellulose-gel. PAAm/cellulose-gel was then immersed in an 80% (v/v) ethanol aqueous solution at room temperature for 12 h to obtain PAAm/cellulose-elastomer.

### Fabrication of PAAm/diatomite-elastomer

The prepared AM monomer solution and 2 wt.% diatomite suspension were mixed at a weight ratio of 12:5 and stirred at a high speed on an orbital shaker for 1 h. The resulting suspension was transferred into molds and degassed in a vacuum oven at 20 °C under a vacuum of −0.04 MPa for 10 min to remove air bubbles. The molds were subsequently placed in an oven at atmospheric pressure and 65 °C for 2 h to complete the polymerization, obtaining PAAm/diatomite-gel. PAAm/diatomite-gel was then immersed in an 80% (v/v) ethanol aqueous solution at room temperature for 12 h to obtain PAAm/diatomite-elastomer.

## Data Availability

The data supporting the findings of this work are available within the article and its supplementary files. All data are available from the corresponding authors upon request.
