# Supplementary material for: A Bioinspired Cellulosic Elastomer with Ultrahigh Robustness Enabled by Structure-Induced Reconstruction for Impact Protection
Source: Research (Wash D C). 2026 Oct 8;9:1460. doi: 10.34133/research.1460 (PMC13646459; doi:10.34133/research.1460)
Supplement: Supplementary file 1 [file research.1460.f1.docx]

**Supplementary Information**

**A Bioinspired Cellulosic Elastomer with Ultrahigh Robustness Enabled by Structure-Induced Reconstruction for Impact Protection**

Geyuan Jiang^1,2^, Minxin Wang^2^, Guangwen Xu^1,2,*^, Dawei Zhao^2,3,*^, and Haipeng Yu^3,*^

1. School of Materials Science and Engineering, Shenyang University of Technology, Shenyang, Liaoning, China.
2. Key Laboratory on Resources Chemicals and Materials of Ministry of Education, Shenyang University of Chemical Technology, Shenyang, Liaoning, China.
3. Key Laboratory of Bio-based Material Science and Technology of Ministry of Education, Northeast Forestry University, Harbin, Heilongjiang, China.

* Corresponding: daweizhao@syuct.edu.cn; [yuhaipeng20000@nefu.edu.cn](mailto:yuhaipeng20000@nefu.edu.cn); gwxu@syuct.edu.cn

**Supplementary Information File Includes:**

Methods

Figures S1 to S30

Tables S1 to S2

References

**Methods**

**Characterization**

The Scanning electron microscopy microstructure of specimens was characterized by a JSM-IT800 microscope (JEOL, Tokyo, Japan) at an operating voltage of 10 kV. The FTIR spectra of specimens were obtained by a Nicolet IS50 FTIR instrument (Thermo Fisher Scientific Inc., Waltham, MA, USA). All spectra were measured in ATR mode, with data recorded in the range of 500-4000 cm^-1^. Solid-state nuclear magnetic resonance hydrogen spectroscopy was performed at 25 ℃ using a Bruker 600M (Bruker, Germany). The ^1^H NMR spectra were acquired via dipole-dipole magic angle spinning (DD MAS) with scans. Rheology measurements were conducted on a MCR92 hybrid rheometer by using the parallel plate geometry with an angular frequency ranging from 10^-2^ to 10^2^ rad/s (Anton Paar, Austria).

**Small-angle x-ray scattering and Wide-angel x-ray scattering**

To analyze the crystal structure and spacing of B-gel, PAAm-elastomer, and B-elastomer, 2D Small-angle x-ray scattering (SAXS) and Wide-angel x-ray scattering (WAXS) techniques were employed using a Xeuss 3.0 SAXS/WAXS system, equipped with an Eiger2R 1M detector, which has a pixel size of 75 μm. The x-ray source used was a copper target (Cu-Kα) operating at 8.05 keV with a wavelength of 1.54189 Å. The experiment was conducted under a vacuum environment (<1 mbar). The specimen-to-detector distance was set to 1000 mm and 100 mm to ensure optimal scattering resolution. The scattering intensity was corrected for water and air background, and the data were analyzed using scattering vector and azimuthal angle coordinates. WAXS assessed inter-crystal spacing (d). The inter-crystal spacing (d) of material was quantified by the 1D scattering curve of corrected scattering intensity (Iq^2^) versus scattering vector (q) obtained as follows:

$d = \frac{2\pi}{q}$	(1)

**Finite element analysis**

Finite element analysis (FEA) was carried out using COMSOL Multiphysics (version 6.3) to quantify the stress transfer and dissipation capability of the biomimetic structure in B-elastomer under tensile and compressive loading. A mechanical model was constructed with boundary conditions applied along the X, Y, and Z directions to calculate the stress distribution within the B-elastomer and its biomimetic framework. The simulations were based on Hooke's law, while nonlinear mechanical behavior was based on Green-Lagrange finite strain theory.

**Mechanical properties test**

The mechanical properties of B-elastomer, PAAm-elastomer, PAAm/cellulose-elastomer, PAAm/diatomite elastomer, and several commercial elastomers were evaluated using an electronic universal testing machine (UTM2503, SUNS, China) equipped with a 5000 N sensor. For the compression test, cylindrical specimens with a diameter of 20 mm were tested at a constant rate of 10 mm min^-1^, which was appropriate for all compression tests. For load-unload compression tests, cylindrical specimens were subjected to a strain from 10% to 90% and for cyclic compression tests, the specimens were stretched to 70% strain at room temperature and cycled continuously for 50 cycles.

For the uniaxial tensile tests, the specimens were molded into dumbbell-shaped samples (with dimensions according to ISO-37) until the specimens reached ultimate failure. All tensile tests were conducted at a constant speed of 50 mm/min unless otherwise noted. In load-unload tensile tests, the dumbbell-shaped specimens were stretched from 10% to 100%, followed by an immediate return to the initial position at the same speed.

Young's modulus and compression modulus were determined from the initial slope of the stress-strain curve in the 5%-10% strain range, and toughness was calculated by integrating over the area of the stress-strain curve. The corresponding energy dissipation and dumping capacity were calculated by integrating the area between the loading and unloading curves:

$Energy dissipation = \int\sigma d\varepsilon$	(2)

$Dumping capacity = \frac{\int_{loading} \sigma d\varepsilon-\int_{unloading} \sigma d\varepsilon}{\int_{loading} \sigma d\varepsilon}$	(3)

Where $\sigma$ and $\varepsilon$ were the stress and strain, respectively.

**Puncture-resistance tests**

In puncture mode, a puncture resistance test was conducted via a tensile testing machine (UTM2503, SUNS, China) with a load sensor of 5 kN. A piece of elastomer specimens (with thickness of approximately 3 mm) was fixed horizontally on a cylindrical stainless-steel frame, and loaded on the tensile tester. A penetrating probe (cone-shaped, blunt-tip, with radius of 0.5 mm and diameter of 1 mm) was programmed to move vertically at the speed of 50 mm/min before piercing into the elastomer. The force and displacement of the probe were recorded during the puncture process until the elastomer broke. The puncture energies (E) were calculated from the curves of the puncture force (F) and puncture displacement (D) via the following equation:

$E = \int_{0}^{D_{max}} FdD$	(4)

where D_max_ is the maximum displacement before penetration.

**Split-Hopkinson pressure bar experiment**

A split-Hopkinson pressure bar (SHPB) experiment (ALT1000, ARCHIMED ES, China), which includes an air pump, strike bar, incident bar, transmission bar, absorber bar, and a data acquisition system, was used to test the dynamic impact properties of B-elastomer during high-speed impact. All the specimens were 40 mm in diameter and 20 mm thick, meeting the 1:2 aspect ratio criterion. The striking rod was accelerated and fired via a nitrogen pump, and an elastic compression wave was generated when the striking rod collided with the incident rod at the time of collision. When the incident wave propagated to the junction of the incident rod and B-elastomer, owing to the impedance inconsistency between the two, a part of it formed a reflected wave, and the remaining part was transmitted through B-elastomer to form a transmitted wave, which eventually dissipated. The data acquisition system then captured the incident, reflected, and transmitted pulses, which were subsequently analyzed to determine the mechanical properties of B-elastomer. The wave signals were derived from theoretical equations pertaining to one-dimensional stress waves. The stress (σ(t)), strain rate (ε^·^(t)), and strain history (ε(t)) of the specimens can be determined by the following equations:

${\sigma(t) =E}_{0} \frac{A}{A_{0}} \varepsilon_{t}\left( t \right)$	(5)

$\varepsilon(t) = -2 \frac{C_{0}}{L}\varepsilon_{r}\left( t \right)$	(6)

$\varepsilon(t) = -2 \frac{C_{0}}{L}\int_{0}^{t} \varepsilon_{r}\left( t \right)dt$	(7)

where *E_0_* was the elastic modulus of the incident bar, *A* and *A_0_* represented the cross-sectional areas of the specimen and the incident bar, respectively. *L* denoted the original length of the specimen, while C_0_ indicated the speed of the elastic wave in the bar. The terms *ε_r_(t)* and *ε_t_(t)* referred to the reflected and transmitted strain pulses, respectively. SHPB impact toughnesses (*W_impact_*) were defined as the energy absorption density during the loading phase, calculated as the area under the engineering stress–strain curve:

𝑊_𝑖𝑚𝑝𝑎𝑐𝑡_ = $\int_{0}^{\varepsilon_{\mathrm{peak}}} \sigma\left( \varepsilon\right)d\varepsilon$	(8)

Where ε_peak_ represents the strain at the peak engineering stress.

**Falling-Ball impact testing of B-elastomer**

To evaluate the protective performance of B-elastomer, specimens of identical size were placed on a glass slide as a protective layer, alongside several commercial elastomers for comparison. An iron ball weighing 256 g was dropped freely from a fixed height of 70 cm, directly impacting the hydrogel-covered glass slide. This test was conducted to assess the ability of B-elastomer to absorb impact energy and protect the underlying glass.

**Measurement of self-healing ability**

To evaluate the self-healing capability of B-elastomer, two specimens of similar size were brought into contact in the healing solution at room temperature for 24 h, after which their tensile and compressive performance was measured.

**Impact buffering performance tests**

The impact buffering performance tests of the elastomers was evaluated using a vertical impact configuration. A spherical projectile was launched under fixed conditions to ensure consistent input, and two force sensors were aligned above and below the specimen to capture the transient stress response.

To minimize the influence of impact variability and system offsets, a reference baseline was established from repeated blank measurements without a specimen. For each test, the stress difference between the bottom and top sensors was defined as $\Delta\sigma$, and the averaged value of the blank tests ($\bar{\Delta\sigma}_{\text{blank}}$) was used as the reference baseline.

$\Delta\sigma=\sigma_{\text{out}}-\sigma_{\text{in}}$	(9)

For each elastomer, the corresponding averaged stress difference ($\bar{\Delta\sigma}_{\text{sample}}$) was obtained under identical conditions. The impact response was then quantified by a dimensionless attenuation efficiency:

$\eta_{\text{attenuation efficiency}}=1-\frac{\bar{\Delta\sigma}_{\text{sample}}}{\bar{\Delta\sigma}_{\text{blank}}}$	(10)

which reflects the relative stress transmission of the system. All samples were tested at identical thickness of 3 mm to exclude geometric effects.

**Cytotoxicity test for human fibroblasts**

The cytotoxicity of B-elastomer extract liquid was evaluated using the CCK-8 assay. Extract liquids of B-elastomer at various concentrations (12.5, 25, 50, 100, and 200 μg/mL) were prepared by immersing them into complete media 24 h after sterilizing under UV irradiation. ‌Normal human dermal fibroblasts (NHDF) cells (4 × 10^3^ cell/well) were first seeded in 96-well plates and incubated with DMEM for 24 h (37 ℃, 5% CO_2_). After removing the culture medium, the unattached NHDF cells were rinsed with PBS buffer solution. Then, extract liquid with diverse concentrations was incubated with NHDF cells for another 24 h. Finally, 10 μL of a CCK-8 solution was added to wells containing cells and incubated together for 2 h. The cells treated without B-elastomer extract were applied as a blank control. The absorbance value at 450 nm was measured using an enzyme immunoassay analyzer.

The cell viability of the plastic is calculated as shown in the equation:

$Cell viability = [\frac{As ― Ab}{Ac ― Ab}]\times100\%$	(11)

Among them, *As* is the test hole absorbance, *Ac* is the control hole absorbance, *Ab* is the white hole absorbance.

The cytotoxicity test was conducted using the live/dead staining to detect the viability changes of NHDF cells. Extract liquids of B-elastomer at various concentrations (12.5-200 μg/mL) were prepared by immersing them into complete media 24 h after sterilizing under UV irradiation. NHDF cells (2×10^4^ cell/well) were first seeded in confocal dish and incubated with DMEM for 24 h (37 ℃, 5% CO_2_). After removing the culture medium, the unattached NHDF cells were rinsed with PBS buffer solution. Then, extract liquid with diverse concentrations was incubated with NHDF cells for another 24 h. Before staining, the adherent cells were gently washed with PBS, the supernatant was removed, and the active esterase contained in the petri dish was removed. Add enough solution (2 μM Calcein-AM and 8 μMPI) and the cells were incubated at room temperature for 30-45 min away from light. Sucking out the dye work solution to terminate incubation. The cells treated without B-elastomer extract were applied as a blank control. The stained cells were observed under confocal microscope.

**Determination of the degree of polymerization of cellulose**

The degree of polymerization of cellulose was determined by the viscosity method according to the Chinese national standard GB/T 5881-2024 using a copper ethylenediamine solution and an Ubbelohde viscometer.

**Figures**

**Fig. S1.** (A) The mean value of the radius of gyration in PAAm-gel, PAAm-elastomer, B-gel and B-elastomer. (B)The mean value of solvent-accessible surface area of PAAm molecules in PAAm-gel, PAAm-elastomer, B-gel and B-elastomer.

**Fig. S2.** G''/G' (tanθ) curves compared between B-gel and B-elastomer.

**Fig. S3.** (A) The WAXS 2D patterns of PAAm-elastomer. (B) The WAXS curves between PAAm-elastomer and B-elastomer.

**Fig. S4.** Comparison of SAXS 2D patterns among (A) B-gel, (B) PAAm-elastomer, and (C) B-elastomer.

**Fig. S5.** Comparison of SAXS curves for B-gel, B-elastomer, and PAAm-elastomer.

**Fig. S6.** Scanning electron microscopy (SEM) images of B-gel (A) and B-elastomer after ethanol stimulation (B) reveal the transition from a relatively loose porous network to a more compact network morphology.

**Fig. S7.** Investigating the ^1^H SSNMR spectra of (A) B-gel and B-elastomer and (B) PAAm-elastomer and B-elastomer.

**Fig. S8.** Optical images of B‑elastomer showing (A) mechanical twisting ability and (B) compressive resistance.

**Fig. S9.** Investigating the corresponding quantified energy dissipation for B-elastomer under load-unload compressive strains.

**Fig. S10.** Investigating the corresponding quantified energy dissipation of B-elastomer under cyclic compressive strains.

**Fig. S11.** Comparison of compressive stress-strain curves between B-elastomer and several commercial elastomers, including expanded ethylene propylene diene monomer (EEPDM), expanded fluoroelastomer (EFKM), expanded nitrile butadiene rubber (ENBR), expanded silicone rubber (ESiR), expanded polyurethane (EPU), expanded chloroprene rubber (ECR), expanded chloroprene rubber (EPE).

**Fig. S12.** Comparison of compression strength and compressive modulus between B-elastomer and several commercial elastomers.

**Fig. S13.** Tensile stress-strain curves of B-elastomer and several commercial elastomers.

**Fig. S14.** Comparison of the tensile strength and tensile modulus of B-elastomer and several commercial elastomers. Data in Supplementary Figure 12 are expressed as mean values ± standard deviation (*n* = 3).

**Fig. S15.** Compressive stress distribution nephograms of PAAm-elastomer and B-elastomer under compressive strains ranging from 1% to 60% obtained from FEA.

**Fig. S16.** (A) Schematic illustration of the positions for evaluation points in compression models of PAAm-elastomer, Cellulose fibers in B-elastomer, Diatomite in B-elastomer, and B-elastomer obtained from FEA. (B) Comparison of the overall compressive stress-strain curves for the PAAm-elastomer and B-elastomer.

**Fig. S17.** Tensile stress distribution nephograms of PAAm-elastomer and B-elastomer under tensile strains ranging from 1% to 30% obtained from FEA.

**Fig. S18.** (A) Schematic illustration of the positions for evaluation points in tensile models of PAAm-elastomer, Cellulose fibers in B-elastomer, Diatomite in B-elastomer, and B-elastomer obtained from FEA. (B) Comparison of the overall tensile stress–strain curves for the PAAm-elastomer and B-elastomer.

**Fig. S19.** Comparison of puncture force for B-elastomer with several reported elastomers[1–8].

**Fig. S20.** Comparison of corresponding maximum stress (A) and impact toughness (B) for B-elastomer at different incident speeds.

**Fig. S21.** SHPB validity verification for B-elastomer at the speed rate of 30m s^-1^. (A) Representative raw SHPB voltage signals for B-elastomer. (B) Incident (ε_i_), reflected (ε_r_), and transmitted (ε_t_) strain histories. The specimen aspect ratio was controlled to L/D = 1:2 to minimize inertial effects.

**Fig. S22.** Optical images and schematic diagrams showing the self-healing capability of B-elastomer.

**Fig. S23.** Compressive stress-strain curves of the B‑elastomer before and after self‑healing, with an inset showing that the self-healed B‑elastomer can still be compressed to 95% strain and fully recover.

**Fig. S24.** Investigating the peel force of the B-elastomer.

**Fig. S25.** SEM images of the ultrasonically fibrillated cellulose at (A) 30,000× and (B) 50,000× magnifications.

**Fig. S26.** SEM images of nano-diatomite at (A) 3,000× and (B) 30,000× magnifications, showing the microscale diatomite skeleton with abundant nanoscale pores.

**Fig. S27.** Effect of cellulose dispersion and diatomite suspension concentrations on the suspension stability of different formulations. (A) Optical images of formulations prepared with different cellulose dispersion and diatomite suspension concentrations. (B) Sedimentation behavior of the corresponding formulations after standing for 30 min, with the red dashed boxes highlighting the sedimented regions. In the figure, “cel” and “dia” denote cellulose dispersion and diatomite suspension, respectively. The percentages indicate the concentrations of the corresponding cellulose dispersion and diatomite suspension, rather than their weight fractions in the final elastomer. The weight ratio of AM monomer solution/cellulose dispersion/diatomite suspension was maintained at 24:5:5 for all formulations.

**Fig. S28.** Tensile stress–strain curves of elastomers prepared with different cellulose dispersion and diatomite suspension concentrations. In the figure, “cel” and “dia” denote cellulose dispersion and diatomite suspension, respectively. The percentages indicate the concentrations of the corresponding cellulose dispersion and diatomite suspension, rather than their weight fractions in the final elastomer. The weight ratio of AM monomer solution/cellulose dispersion/diatomite suspension was maintained at 24:5:5 for all formulations.

**Fig. S29.** Effect of ethanol concentration on the appearance and tensile properties of B-elastomer. (A) Optical images of B-elastomers obtained by immersing B-gel in ethanol aqueous solutions with concentrations ranging from 0% to 100% (v/v) at room temperature for 12 h. (B) Volume change ratio of B-elastomers obtained by immersing B-gel in ethanol aqueous solutions with varying concentrations from 0% to 100% (v/v) at room temperature for 12 h. (C) Tensile stress–strain curves of B-elastomers with pronounced shrinkage after immersion in 60%–100% (v/v) ethanol aqueous solutions at room temperature for 12 h. Based on the combined results of Fig. S25, 80% (v/v) ethanol aqueous solution was selected as the optimal concentration.

**Fig. S30.** Effect of immersion time on the tensile stress–strain behavior of B-elastomer in 80% (v/v) ethanol solution. Tensile stress–strain curves of B-elastomers after different immersion durations at room temperature. The mechanical properties approached stabilization after 10 h, and 12 h was selected as the optimal immersion time to ensure sufficient structural and mechanical stabilization.

**Tables S1 to S2**

**Table S1.** Price average estimation of B-elastomer for 5 preparations. Each preparation reused only ethanol, with 20% wt.% added as compensation.

| **Feedstocks** | **Price**  **($/t)** | **Consumption**  **(t)** | **Cost**  **($)** | **Source** |
| --- | --- | --- | --- | --- |
| Cellulose | 250 | 0.01 | 2.45 | https://www.alibaba.com/product-detail/Hot-Sales-Bleached-Hardwood-Kraft-Pulp_1600917310137.html?spm=a2700.galleryofferlist.normal_offer.d_title.794013a0V9ryIn&priceId=78d3ddde0a8340d298aa987726be454d |
| Acrylamide | 1000 | 0.39 | 391.83 | https://www.alibaba.com/product-detail/Hot-Selling-Acrylamide-Manufacturers-AM-79061_1601407024941.html?spm=a2700 |
| Ammonium persulfate | 5731.32 | 0.002 | 11.23 | https://www.aladdin-e.com/zh_cn/a498725.html |
| N,N ′ -methylenebis | 26142.18 | 0.0004 | 10.24 | https://www.aladdin-e.com/zh_cn/m104022.html |
| Diatomite | 230 | 0.01 | 2.26 | https://www.alibaba.com/product-detail/Food-Grade-Industrial-Grade-325-Mesh_1601320485995.html?spm=a2700.galleryofferlist.topad_classic.d_title.a34d13a0YvhONr&priceId=69c6eaf808d24f789e51776ef9a6a1ee |
| H_2_O | 0.33 | 2.92 | 0.96 | https://scj.shenyang.gov.cn/zwgk/fdzdgknr/gsgg/202503/t20250331_4829895.html |
| Ethanol | 800 | 2.01 | 1607.48 | https://chinese.alibaba.com/product-detail/Ethanol-Alcohol-Perfume-Used-High-Quality-10000014052937.html?spm=a27aq |
| Electricity | 0.0924 | 695.90 | 64.30 | https://scj.shenyang.gov.cn/zwgk/fdzdgknr/gsgg/202503/t20250331_4829895.html |
| Total cost | 2091 | | | |

**Table S2.** Comparative prices among ESiR, EPU, ECR, EPE, and B-elastomer.

| **Materials** | **Price**  **($/t)** | **Source** |
| --- | --- | --- |
| ESiR | 2900 | https://www.alibaba.com/product-detail/Soft-Medium-Firm-Hardness-Sponge-Custom_1601624157054.html?spm=a2700.galleryofferlist.p_offer.d_title.6b7413a0w7JN3W&priceId=a853d57e30ea48799b3244b7c760d89e |
| EPU | 1580 | https://www.alibaba.com/product-detail/PORON-Shock-absorbing-Foam-Pu-Light_1601664192102.html?spm=a2700.galleryofferlist.normal_offer.d_title.321a13a08vHFe9&priceId=535bc8da2fc2494d80a9ad07874b555e |
| ECR | 2195 | https://www.alibaba.com/product-detail/Custom-Cut-Neoprene-CR-Closed-Cell_1601397030853.html?spm=a2700.galleryofferlist.normal_offer.d_title.543513a0cuy21D&priceId=f33a4be5de69450da2185e243dfbedba |
| EPE | 2617 | https://www.alibaba.com/product-detail/Factory-Custom-Eco-Friendly-Waterproof-EPE_1601463805264.html?spm=a2700.galleryofferlist.normal_offer.d_title.fecf13a0ru3kCb&priceId=54b0f494adfd4555b74c8176747571e8 |
| B-elastomer | 2091 | This work |

**REFERENCES**

1.	Tan Y, Wang Y, Zhang P, Li J, Wang F, Shan L, Guo J, Wang Z, Liu J. Bioinspired hierarchical hydrogels engineered with extreme impact resistance. *Adv Funct Mater.* 2025;35:e08034.

2.	Chen S, Wu K, Gao H, Sun X, Zhang S, Li X, Zhang Z, Wen S, Zhu Y, Wu H, et al. Biomimetic discontinuous Bouligand structural design enables high-performance nanocomposites. *Matter*. 2022;5:1563–1577.

3.	Chen X, Zhong Q, Cui C, Ma L, Liu S, Zhang Q, Wu Y, An L, Cheng Y, Ye S, et al. Extremely tough, puncture-resistant, transparent, and photoluminescent polyurethane elastomers for crack self-diagnose and healing tracking. *ACS Appl Mater Interfaces*. 2020;12:30847–30855.

4.	Hou K, Zhao S, Wang D, Zhao P, Li C, Zuo J. A puncture-resistant and self-healing conductive gel for multifunctional electronic skin. *Adv Funct Mater*. 2021;31:2107006.

5.	Qu S, Liu G, Liu Y, Yang M, Yang L, Deng J, Bai R, Wang C, Wang Y, Ding Y, et al. Engineering energy dissipation pathway via topological design: toward tunable macroscopic mechanical performances of dynamic polymer networks. *Angew Chem Int Ed*. 2025;64:e202513613.

6.	Tang B, Hu J, Zhao Z, Li S, Lv H, Yang X. Puncture-resistant hydrogels with high mechanical performance achieved by the supersaturated salt. *Mater Horiz*. 2025;12:4229–4237.

7.	Yuan S, Feng Y, Wang H, Chen S, Li J, Zhu Y, Yu S, Wang Z. Sword and board in one: a bioinspired nanocomposite membrane for guided bone regeneration. *Adv Mater*. 2025;37:2504577.

8.	Zhang Z, Sang M, Li Z, Pan Y, Wu J, Duan S, Gong X. In-situ localised alignment assisted salting-out enhanced ionogels with high strength, toughness and impact resistance. *Nat Commun*. 2025;16:7683.
